# Effect of finger–ball friction on upper limb movement during fastball pitching in baseball

**DOI:** 10.1038/s41598-025-12298-8

**Published:** 2025-07-30

**Authors:** Takeshi Yamaguchi, Sota Suzuki, Shinnosuke Suzuki, Toshiaki Nishi, Takehiro Fukuda, Daiki Nasu

**Affiliations:** 1 Graduate School of Engineering, Tohoku University, 6-6-1 Aramaki-Aza-Aoba, Aoba-Ku, Sendai, Miyagi 980-8579 Japan; 2 Graduate School of Biomedical Engineering, Tohoku University, 6-6-1 Aramaki-Aza-Aoba, Aoba-Ku, Sendai, Miyagi 980-8579 Japan; 3 NTT Communication Science Laboratories, NTT Corporation, 3-1 Morinosato-Wakamiya, Atsugi, Kanagawa 243-0198 Japan

**Keywords:** Baseball, Pitching, Friction, Upper limb motion, Centrifugal force, Biomedical engineering, Motor control, Sensory processing, Biomechanics

## Abstract

This study investigated the effect of the friction between the ball and fingertips (finger–ball friction) on upper limb movement during four-seam fastball pitching in terms of the centrifugal force acting on the ball. Eight skilled pitchers threw four-seam fastballs at approximately 130 km/h toward a target behind the home base. Water was applied as a low-friction condition and rosin powder was applied as a high-friction condition between the fingertips and the ball. Hand velocity and pitching radius (i.e., radius of the motion trajectory of the hand) were calculated from motion capture data. Centrifugal force evaluation index was calculated as the square of hand velocity divided by the pitching radius. Statistical parametric mapping was performed to compare the time-series of each variable between foot contact and ball release. Although no significant differences were observed, a tendency for hand velocity to decrease under low-friction conditions during the acceleration phase was observed. Additionally, the pitching radius was significantly greater under low-friction conditions around the maximum shoulder rotation timing, and consequently, the centrifugal force index significantly decreased during the first half of the acceleration phase. These findings deepen our understanding of how pitchers adjust their throwing motion under different finger–ball friction conditions.

## Introduction

Baseball pitching requires advanced motion control to set the trajectory of the ball while delicately manipulating its rotation using the fingertips^1,2^. Friction between the fingers and the ball (i.e., finger–ball friction) ensures a proper grip during pitching until the moment of release, at which point the tangential force increases sharply as the ball rolls, generating spin^3^. Particularly, in a fastball pitch, an increase in the tangential force component of the finger force contributes to an increase in the ball spin rate^4^. Therefore, pitchers need to adjust to changes in finger–ball friction, such as when these get wet due to sweat or rain. To increase friction, grip-enhancing agents such as rosin powder or rubbing mud are applied to the fingertips or ball^5–7^. In baseball, many studies have investigated the relationship between the paramters of ball release (BR) and pitching performance^8–10^, as well as on the forces acting on the ball from the fingertips during its release^3,11,12^. However, the influence of finger–ball friction during BR on pitching performance and pitching motion remains a topic of further investigation.

In a previous study, we demonstrated that various finger–ball friction conditions affect the slip distance during BR in fastball pitching, thereby significantly affecting the ball spin rate and pitch control^13^. Specifically, wetting the ball and fingertips with water increased the slip distance between the fingertips and ball, resulting in a lower ball spin rate compared to when rosin powder was applied. Additionally, there was also a tendency for the ball to be thrown upward and laterally, thereby decreasing pitch control. In that study, participants were instructed to pitch at the same ball speed (130 km/h) under different finger–ball friction conditions. The ball speed was significantly lower under low friction (simulated by applying water) compared to high friction (simulated by applying rosin powder). This has been attributed to the participants’ perception of the slippage between the fingertips and ball, causing them to adjust their pitching motion.

During baseball pitching, a centrifugal force acts on the ball due to rotational motion of the upper limb^14^. This centrifugal force is inversely proportional to the pitching radius (i.e., the radius of ball motion trajectory during pitching) and is proportional to the square of the ball translation velocity. During pitching, the ball is subjected not only to a normal force directed toward its center but also to a tangential (frictional) force exerted by the fingertips (thumb, index, and middle finger)^3,11^, which can cause the ball to slip. To prevent this, either the centrifugal forces need to be reduced, or the forces acting on the ball from the fingertips need to be increased. Reducing the ball velocity by wetting the ball, as described above, is one possible strategy for reducing the centrifugal force acting on the ball. Additionally, the pitching radius could have also been increased under low friction, since this can also reduce the centrifugal force acting on the ball.

We hypothesized that, under slippery conditions with low finger–ball friction, the centrifugal force acting on the ball is reduced, because the velocity and/or trajectory of the hand adjusts during pitching. Accordingly, this study investigated the relationship between the centrifugal force acting on the ball and finger–ball friction by determining the hand velocity and pitching radius from the trajectory of fingertip motion.

## Results

Eight semiprofessional baseball pitchers were instructed to throw four-seam fastballs toward a target placed behind the home plate at a velocity of 130 km/h. Each pitcher threw five times each under low and high-friction conditions, which were simulated by applying water and rosin powder, respectively.

**Fig. 1:**
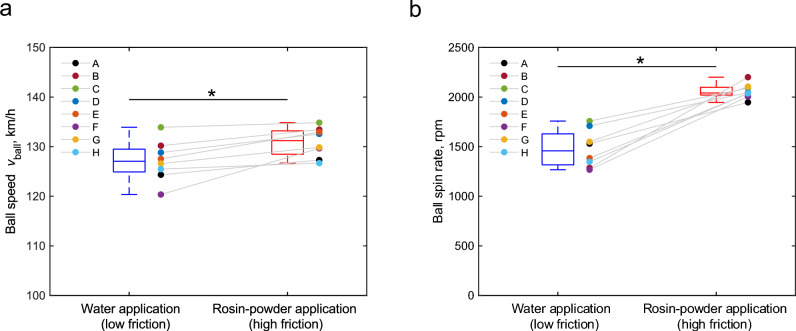
Box plots for (**a**) the ball speed and (**b**) the ball spin rate under low and high-friction conditions. In boxplots, the circles represent the mean values within participants.* indicates *p* < 0.05.

Figure 1 illustrates the boxplots for ball speed and ball spin rate measured with a Doppler radar tracking system under both conditions. Wilcoxon rank sum test revealed that the ball speed (Fig. 1(a), *p* = 0.012, *r* = 0.630) and the ball spin rate (Fig. 1(b), *p* = 0.012, *r* = 0.630) were significanlty lower under low-friction conditions.

**Fig. 2:**
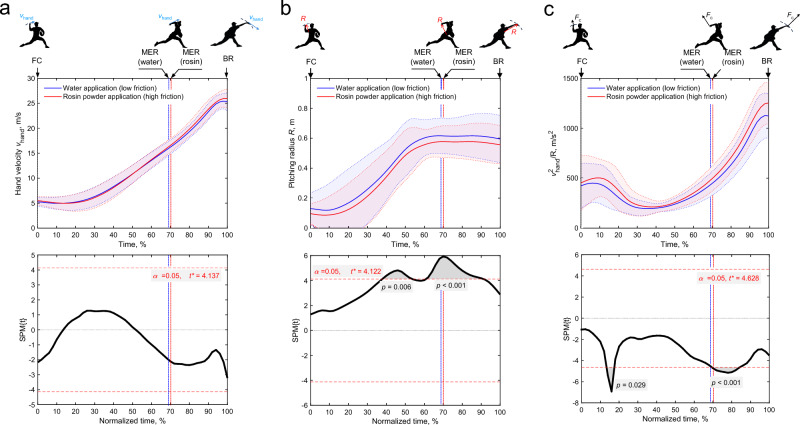
Time-series data of the group mean value and SPM results for (**a**) the hand velocity (*v*_hand_), (**b**) the pitching radius (*R*), and (**c**) the index of centrifugal force (*v*_hand_^2^/*R*). In the time-series graphs in the upper section of each subfigure, the solid line indicates the between-participant mean, and the shaded area indicates the between-participant mean ± standard deviation. Vertical lines shown in the figure represents the average MER time across participants. MER time ranged from 59.2% to 75.5% under low-friction conditions and from 61.2% to 77.6% under high frition conditions, depending on the pitcher. In the SPM results in the lower section of each subfigure, the critical* t*-value threshold is presented in red text and by the dashed lines above and below 0. Where the *t*-value exceeded the threshold, significance was reached and is highlighted by gray-shaded area. FC, foot contact; MER, maximum shoulder external rotation; BR, ball release.

Figure 2 depicts the time-series change in the group mean value and statistical parametric mapping (SPM) two-tailed paired sample *t*-test results for the translational velocity of the hand (*v*_hand_, i.e., the velcoity of the marker attached to the third metacarpal bone), the pitching radius (*R*), and the centrifugal force index (*v*_hand_^2^/*R*) between the leading foot contact (FC, 0% normalized time) and the BR (100% normalized time). The vertical lines present in the figure represents the average maximum shoulder rotation (MER) time across participants. Notably, there was no significant difference in the mean MER time between conditions (*p* = 0.674, *r* = 0.105).

As shown in Fig. 2(a), under both conditions, *v*_hand_ increases toward BR and becomes nearly constant just before BR*.* Although there were no significant defferences in *v*_hand_ among friction conditions during late-cocking and acceleration phases (*p* > 0.05), *v*_hand_ tends to be lower under low-friction conditions during acceleration phase.

As illustrated in Fig. 2(b), under both conditions, *R* increases up to 60% of normalized time then remains nearly constant until the moment of BR. SPM analysis revealed that *R* was significantly greater under low-friction conditions between 38 and 52% (*p* = 0.006) and between 62 and 90% (*p* < 0.001) of the normalized time.

Moreover, as shown in Fig. 2(c), under both conditions, *v*_hand_^2^/*R* initially decreases following FC, and subsequently increases toward BR. SPM analysis revealed that *v*_hand_^2^/*R* was significantly lower under low-friction conditions between 14 and 16% (*p* = 0.029), and between 70 to 84% *(p* < 0.001) of the normalized time.

## Discussion

This study evaluated the upper limb movements of baseball pitchers under conditions of low and high fingertip–ball friction, simulated by applying water and rosin powder, respectively. As previously reported^13^, ball velocity and ball spin rate significantly decreased under low-friction conditions. Specifically, the reduction in spin rate under low-friction conditions suggests that considerable slip occurs between the fingertips and the ball. It is likely that participants adjusted the motion of the upper limb in an effort to reduce this slip. Our results indicated that the hand velocity tended to be lower under low-friction conditions during acceleration phase and the pitching radius was significanlty larger under low-friction conditions before and after the time of MER. Thus, the centrifugal force index was significantly smaller during the first half of the acceleration phase under the low-friction coditions compared to the high-friction conditions. Therefore, under slippery conditions, most participants alter their upper limb movements to reduce centrifugal force by increasing the pitching radius while decreasing hand velocity during the acceleration phase, which confirms our hypothesis. In a questionnaire survey after the experiements, some participants answered that they pitched the ball by pushing their arms forward in the low-friction condition, suggesting that they intentionally performed these movements.

In this study, participants were instructed to maintain a constant pitching velocity of 130 km/h and aim at a target located behind home plate. Therefore, the observed decrease in hand velocity and increase in pitching radius under low-friction conditions may reflect a strategy to suppress slip during the pitching motion while maintaining pitch control, specifically by adjusting the ball’s release velocity and release angles^9,10^. Another possible strategy to suppress slippage is to apply additional fingertip force to generate greater friction, without altering pitching velocity or pitiching radius. In the present study, some pitchers showed little variation in hand velocity and pitching radius across conditions, suggesting they used a strategy other than reducing centrifugal force to suppress slippage. However, doing so can increase the load/fatigue on the forearm muscles, which can result in elbow and other injuries^15^. In line with this, the tightened restrictions on adhesive substances in Major League Baseball led to a decrease in pitching performance^16^ and may have even contributed to an increase in elbow and shoulder injuries^17^. Nevertheless, our findings need to be validated through further studies, particularly through measurements of finger forces^3,11^, finger movements^4,18,19^, and forearm muscle activity^20^ during pitching under different finger–ball friction conditions.

The centrifugal force acting on the ball is a function of hand speed, which is proportional to ball speed. In this study, the ball speed was adjusted to 130 km/h, which is slightly slower than in actual games, to minimize physical burden on the participants. The centrifugal force acting on the ball is estimated to be smaller at slower ball speeds, therefore requiring smaller frictional force to control slippage. In turn, this may require less compensatory movements of the upper limb to reduce centrifugal force. Therefore, the upper limb motion changes observed in the present study may be more pronounced when throwing at higher velocities.

The limitations of this study must be discussed. First, there was only a small number of participants, and only overhand throwers were included. Second, our results were obtained only with four-seam fastball pitches, and thus there is a need to verify whether similar results can be obtained with other types of pitches. Third, no markers were attached to the ball or fingertips, and velocity was calculated using the third metacarpal marker, which could have influenced the measurement of ball velocity, thereby affecting the calculation of centrifugal force. Fourth, although the ball speeds were significantly lower under low friction, different results could have been obtained if the participants were given feedback regarding their ball speed measurements during the experiment as well as in practice.

## Conclusions

This study investigated the effects of low and high finger–ball friction conditions, (simulated by applying water and rosin powder, respectively) on the upper limb movement during four-seam fastball pitching in terms of the centrifugal force acting on the ball. Ball velocity and ball spin rate were found to significantly decrease under low-friction conditions. Under such low-friction conditions, the centrifugal force index was smaller during the first half of the acceleration phase than in the high-friction conditions, mainly due to a increase in pitching radius. These results suggest that most pitchers altered their pitching motion to reduce the centrifugal force acting on the ball to prevent it from slipping. The results of this study deepen our understanding regarding the compensatory arm motions of pitchers when throwing under different finger–ball friction conditions.

## Material and methods

### Participants

This study included 8 semiprofessional male baseball pitchers (6 right-handed, 2 left-handed) from a company baseball league in Japan. The participants had a mean (± standard deviation) age of 28.6 ± 5.1 years, height of 1.78 ± 0.04 m, and body mass of 83.7 ± 5.5 kg. All participants had an overhand throwing motion. The experimental protocol of this study was approved in advance by the Research Ethics Committee of NTT Communication Science Laboratories (R05-13). Each participant was informed of the experimental methods and precautions and provided written informed consent in advance. All experiments were performed in accordance with the Declaration of Helsinki.

### Experimental procedure

**Fig. 3:**
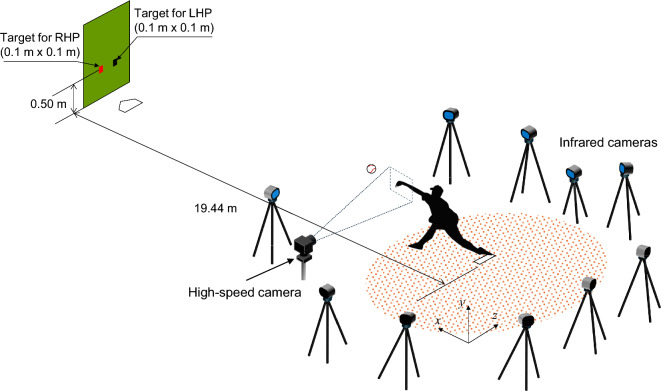
Experimental setup. A participant threw a baseball at a 0.1 × 0.1 m square target in the low outer corner of the strike zone at a distance of 19.44 m (1 m behind home base), which is equivalent to the distance from the mound to the catcher’s mitt. The orange square represents a right-handed pitcher, while the black square represents a left-handed pitcher. RHP, right-handed pitcher; LHP, left-handed pitcher.

The experimental setup and protocols were as descrived in a previous study^13^ and outlined below. Figure 3 shows a schematic diagram of the pitching experiment. Participants were asked to pitch a four-seam fastball at a target in the low outer corner of the strike zone at a distance of 19.44 m (1 m behind home base), which is equivalent to the distance from the mound to the catcher’s mitt. An overhand pitching motion was done with a target speed of 130 km/h, which is slightly slower than in actual games, in consideration of the physical burden on the participants. Participants received feedback regarding the measured ball velocity while practice pitching, but no feedback on pitch velocity was given during the experiment. The baseball (1BJBH10000; MIZUNO Corporation, Tokyo, Japan) used in the experiment had a radius of 37 mm and mass of 145 g. After warming up, the participants first performed the pitching experiment under a no application condition (no application of any agent). Subsequently, participants pitched the ball five times under three different conditions (i.e., applying water, rosin powder, and pine resin) in a random order. Each condition was limited to five pitches in consideration of the effects of fatigue on the pitcher, and thus a total of 20 pitches were performed by each particpant. When applying water, the fingertips and the ball were thoroughly moistened by dipping them in a container of tap water. When applying rosin powder, the participants held the rosin bag and applied a sufficient amount to their fingertips. Pine resin was applied by spraying it on a new ball, and the participants held the ball to apply the substance to their fingertips. After pitching under each condition, an interval of 5 min was given, during which participants thorughly washed and dried their hands, and a new baseball was used at the start of the next condition. During the pitching experiment, a Doppler radar tracking system (Trackman, Vedaek, Denmark) was used to measure ball velocity and ball spin rate. A high-speed camera (MEMRECAM Q2m, NAC Image Technology Co., Ltd.) was used to film the area around the fingers just before the ball was released from the side of the pitcher at 2000 fps. Infrared reflective markers were attached to 39 major joints on the bodies of the participants, and a motion capture system (OptiTrack, Acuity Inc.) was used with 10 infrared cameras (Prime 17W, Acuity Inc.) set up around the mound (Fig. 3). The *x*-axis was defined as the direction from the pitching mound to the home base. The *y*-axis was defined as the vertical direction. The *z*-axis was the direction toward the third base from the mound. The positional coordinates of each marker during the pitching motion were recorded using a motion capture system (OptiTrack, Acuity Inc.) at 360 fps.

### Data analysis

This study analyzed experimental data under low and high-friction conditions, simulated by applying water and rosin powder, respectively. In a previous study, when pine resin was applied, there were no substantial differences in pitching performance across the five trial pitches^13^, likely because participants were not accustomed to pitching under this condition since pine resin is usually prohibited. Thus, this study focused on the application of water and rosin powder only (representing low and high friction, respectively), to ensure clarity and consistency in the analysis.

In this study, the period from the time the leading foot contacted the ground to BR (i.e., the late-cocking and acceleration phases) was normlized to 0%–100%, and the time-series data of variables related to centrifugal force acting on the ball were compared between conditions. Timing of ground contact by leading foot (0% of the normalized time) was defined as the moment when the *y*-coordinates of the marker attached to the heel reached its lowest point after the knee of the leading leg had reached and descend from its peak height. The BR time (100% of normalized time) was determined based on the high-speed camera images^13^. The external rotation (ER) angle of the shoulder was defined as the angle of the forearm to the trunk, calculated based on the method of Miyashita et al.^21,22^, where two corresponding triangles were established between the markers to define the upper body segments. The corresponding triangles for shoulder ER calculations were formed by the markers on the acromion process, elbow joint and spinous process of 10^th^ thoracic vertebrate and those on the wrist, elbow joint and acromion process.

The translational velocity of the hand and the time-series change in the radius of rotation of the hand motion trajectory were determined as described below. The translational velocity of the hand (*v*_hand_) was calculated as the translational velocity of the position coordinates (*x*_TMB_, *y*_TMB_, *z*_TMB_) of the marker attached to the third metacarpal bone using the following formula:

1 $${{v}_{\text{hand}}(t}_{m}) = \frac{\sqrt{{\left({x}_{\text{TMB}}\left({t}_{m + 1}\right)-{x}_{\text{TMB}}\left({t}_{m-1}\right)\right)}^{2} + {\left({y}_{\text{TMB}}\left({t}_{m + 1}\right)-{y}_{\text{TMB}}\left({t}_{m-1}\right)\right)}^{2} + {\left({z}_{\text{TMB}}\left({t}_{m + 1}\right)-{z}_{\text{TMB}}\left({t}_{m-1}\right)\right)}^{2}}}{2 \times \frac{1}{360}}$$

where *t*_m_ is the time in the measured frame *m* of the motion capture data.

For the pitching radius, the radius of curvature at each time point in the movement trajectory of the third metacarpal marker was determined as described below. First, in the time-series data of the third metacarpal marker, 18 frames before and after a certain time (*t*_i_) were taken as a subset. The 3D point cloud in the subset was approximated to a sphere using the least-squares method to obtain the radius of curvature of the sphere, which was used as the pitching radius (*R*). Details regarding the least-squares method are shown in the Appendix.

The centrifugal force (*F*_c_) acting on the ball during pitching is thought to be proportional to the square of the translational velocity of the ball and inversely proportional to the radius of the curvature of the trajectory of the ball. However, since the position of the ball was not measured, *v*_hand_^2^/*R* was used as an index to evaluate the magnitude of the centrifugal force acting on the ball.

**Fig. 4:**
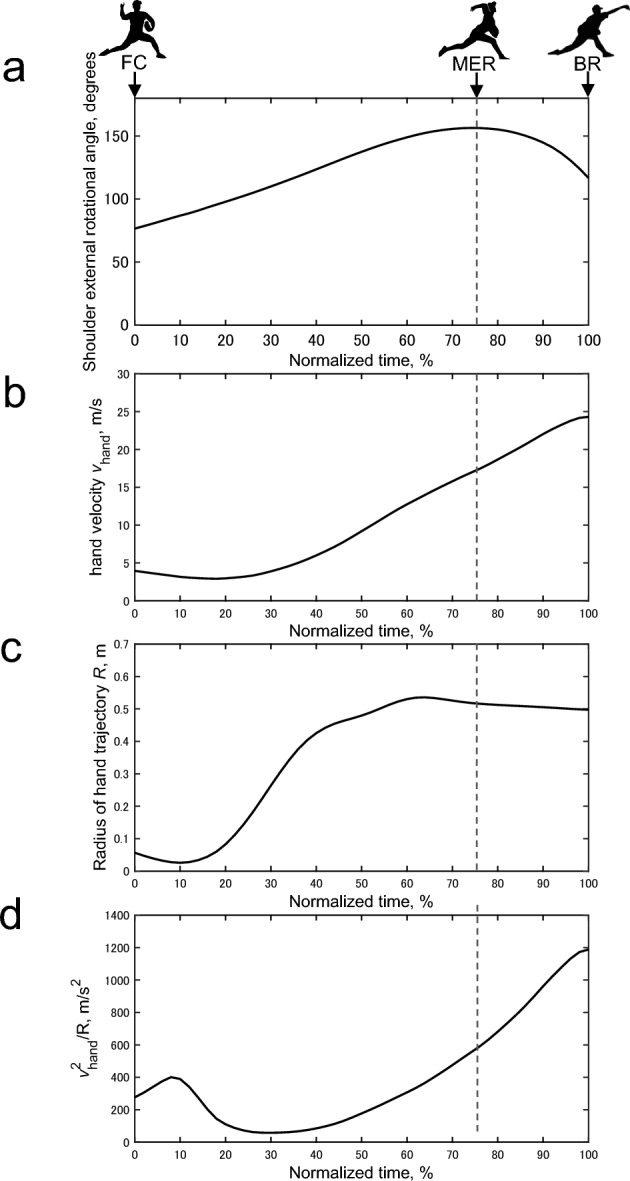
Sample time-series changes in (**a**) shoulder external rotation angle, (**b**) hand velocity (*v*_hand_), (**c**) pitching radius (*R*), and (**d**) the index of centrifugal force (*v*_hand_^2^/*R*) during the late-cocking and acceleration phases under high friction. FC, foot contact; MER, maximum shoulder external rotation; BR, ball release.

Figure 4 shows an example of the time-series changes in the shoulder ER angle, *v*_hand_, *R*, and *v*_hand_^2^/*R* during the late-cocking and acceleration phases under high-friction simulated by applying rosin powder. These analyses were performed using MATLAB (MathWorks, Natick, MA, USA).

### Statistical analysis

Wilcoxon rank sum test was performed to determine differences in the ball speed, spin rate, and MER time under high and low-friction conditions using SPSS Statistics for Windows, Version 19.0 (IBM Corp., Armonk, NY, USA). Pearson’s correlation coefficient *r* was determined as the effect size with values of < 0.3, 0.3–0.5, and > 0.5 indicating small, moderate, and large effects, respectively^23^. Differences in the *v*_hand_, *R*, and *v*_hand_^2^/*R* across the time-series data were examined using one-dimenstional SPM two-tailed paired *t*-tests uisng open source code (www.spm1d.org)^24^. Statistical significance for all statistical anlayzes was set at *p* of < 0.05.

### Appendix. Approximation of the pitching radius

Assuming that the position vector of the center of the sphere with radius (*R*) is ***m*** and the vector of the point cloud is ***v***_**k**_, the residual sum of squares (*C*) can be expressed using the following equation:

A1 $$C = \sum_{k = 1}^{n}{\left\{{\left({{\varvec{v}}}_{\mathbf{k}}-{\varvec{m}}\right)}^{2}-{R}^{2}\right\}}^{2}$$

To approximate the point group (***v***_**k**_) to a sphere, we need to find *R* and ***m*** such that the residual sum of squares *C* is minimized. Therefore, the partial differentiation of the residual sum of squares *C* with respect to *R* and ***m***, respectively, yields *R* and ***m*** such that the partial function obtained is zero. The partial differentiation of *C* by *R* yields the following:

A2 $$\frac{\partial C}{\partial R} = -4R\sum_{k = 1}^{n}\left\{{\left({{\varvec{v}}}_{\mathbf{k}}-{\varvec{m}}\right)}^{2}-{R}^{2}\right\} = 0$$

Since the radius (*R*) is not zero, it is expressed using the following equation.

A3 $$R = \sqrt{ \frac{1}{n}\sum_{k = 1}^{n}{\left({{\varvec{v}}}_{\mathbf{k}}-{\varvec{m}}\right)}^{2}}$$

Differentiating the residual sum of squares *C* by **m** yields:

A4 $$\frac{\partial C}{\partial {\varvec{m}}} = \sum_{k = 1}^{n}\left({{\varvec{v}}}_{\mathbf{k}}-{\varvec{m}}\right)\left\{{\left({{\varvec{v}}}_{\mathbf{k}}-{\varvec{m}}\right)}^{2}-{R}^{2}\right\} = 0$$

When substituting Equation (A3) for *R* in Equation (A4), the following equation is obtained:

A5 $$\overline{{{\varvec{v}} }_{\mathbf{k}}^{3}}-\overline{{{\varvec{v}} }_{\mathbf{k}}}\left(\overline{{{\varvec{v}} }_{\mathbf{k}}^{2}}\right) = 2\left\{\frac{1}{n}\sum_{k = 1}^{n}{{\varvec{v}}}_{\mathbf{k}}\left({{\varvec{v}}}_{\mathbf{k}}\bullet {\varvec{m}}\right)-\overline{{{\varvec{v}} }_{\mathbf{k}}}({\varvec{m}}\bullet \overline{{{\varvec{v}} }_{{\varvec{k}}}})\right\}$$

where, $$\frac{1}{{\varvec{n}}}\sum_{{\varvec{k}}\boldsymbol{ }=\boldsymbol{ }1}^{{\varvec{n}}}{{\varvec{v}}}_{\mathbf{k}}=\overline{{{\varvec{v}} }_{\mathbf{k}}}$$**,** $$\frac{1}{{\varvec{n}}}\sum_{{\varvec{k}}\boldsymbol{ }=\boldsymbol{ }1}^{{\varvec{n}}}{{\varvec{v}}}_{\mathbf{k}}^{2}=\overline{{{\varvec{v}} }_{\mathbf{k}}^{2}}$$, and $$\frac{1}{{\varvec{n}}}\sum_{{\varvec{k}}\boldsymbol{ }=\boldsymbol{ }1}^{{\varvec{n}}}{{\varvec{v}}}_{\mathbf{k}}^{3}=\overline{{{\varvec{v}} }_{\mathbf{k}}^{3}}$$.

Equation A5 can be transformed into the following equation:

A6 $${\varvec{A}}\bullet {\varvec{m}}\boldsymbol{ }=\boldsymbol{ }{\varvec{B}}$$

where, $${\varvec{A}} = 2\left(\frac{1}{n}\sum_{k = 1}^{n}{{\varvec{v}}}_{\mathbf{k}}\bullet {{\varvec{v}}}_{\mathbf{k}}^{\mathbf{T}}-\overline{{{\varvec{v}} }_{\mathbf{k}}}\bullet {\overline{{{\varvec{v}} }_{\mathbf{k}}}}^{\mathbf{T}}\right)$$ and $${\varvec{B}}\boldsymbol{ }=\boldsymbol{ }\overline{{{\varvec{v}} }_{\mathbf{k}}^{3}}-\overline{{{\varvec{v}} }_{\mathbf{k}}}\left(\overline{{{\varvec{v}} }_{\mathbf{k}}^{2}}\right)$$.

Equation (A6) can be transformed as follows to obtain the center position ***m*** of the sphere.

A7 $${\varvec{m}}={{\varvec{A}}}^{-1}\bullet {\varvec{B}}$$

From Equation (A3), the radius (*R*) of the sphere can be determined from its center position (***m***).

## Data Availability

The data that support the findings of this study are available from the corresponding author upon reasonable request.
